# Current OCT Approaches Do Not Reliably Identify TCFAs

**DOI:** 10.4172/2155-9880.1000350

**Published:** 2014-12-04

**Authors:** Mark E. Brezinski, Kishore J Harjai

**Affiliations:** 1 Center for Optics and Modern Physics, Brigham and Women’s Hospital, Boston, M.A, USA; 2 Harvard Medical School, Boston, M.A, USA; 3 Department of Electrical Engineering, Massachusetts Institute of Technology, Cambridge, M.A, USA; 4 Richard and Marion Pearsall Heart Hospital, Geisinger Clinic, Wilkes-Barre, PA, USA

**Keywords:** Plaque rupture, Optical coherence tomography: OCT, Thin capped fibroatheroma: TCFA, Acute coronary syndrome: ACS, Myocardial infarction, Lipid, Macrophages, Massachusetts General Hospital Lightlab

## Abstract

It is now clearly established that Thin-Capped Fibroatheromas (TCFAs) lead to most Acute Coronary Syndromes (ACSs). The ability to selectively intervene on TCFAs predisposed to rupture and ACSs would dramatically alter the practice of cardiology. While the ability of OCT to identify thin walled plaques at micron scale resolutions has represented a major advance, it is a misconception that it can reliably identify TCFAs. One major reason is that the ‘diffuse border’ criteria currently used to determine ‘lipid plaque’ is almost undoubtedly from high scattering in the intima and not because of core composition (necrotic core). A second reason is that, rather than looking at lipid collections, studies need to be focused on identifying necrotic cores with OCT. Necrotic cores are characteristic of TCFAs and not lipid collections. Numerous other OCT approaches are available which can potentially accurately assess TCFAs, but these have not been aggressively pursed which we believe likely stems in part from the misconceptions over the efficacy of ‘diffuse borders’.

## Introduction

### Acute coronary syndromes and OCTs ability to characterize high risk plaque

Acute Coronary Syndromes (ACSs) are the leading cause of death in the industrialized world, representing over 25% of all deaths in the US alone [1–3]. The importance of early aggressive intervention can’t be over emphasized. Specific plaques called Thin-Capped Fibroatheromas (TCFAs) lead to most ACSs [4–12]. When these plaques rupture, they release thrombogenic necrotic material into the blood, a clot forms, and the vessel occludes in about 20% of the cases. Detecting TCFAs prior to rupture, particularly risk stratifying those 20% predisposed to ACS progression, is a central focus of cardiovascular research [7–10,12,13]. TFCAs are characterized by a necrotic (lipid-laden) core, a thin intimal cap, and minimal protrusion into the lumen [4–12]. In addition, they have a loss of cap collagen/smooth muscle, immature angiogenesis, and intermittent inflammation. Plaque strain analysis and autopsy data has demonstrated these plaques have caps less than 75 μm in width and a necrotic core which is soft (high strain) making them susceptible to rupture [14–18]. It is a currently held belief that Optical Coherence Tomography (OCT), in its current state, can identify TCFAs reliably [11,19–20]. Studies do strongly support the ability to identify the thin intimal caps [21–22]. However, contrary to currently held beliefs, the evidence is relatively weak it can reliably identify necrotic cores (or even pure lipid cores).

**Figure 1:**
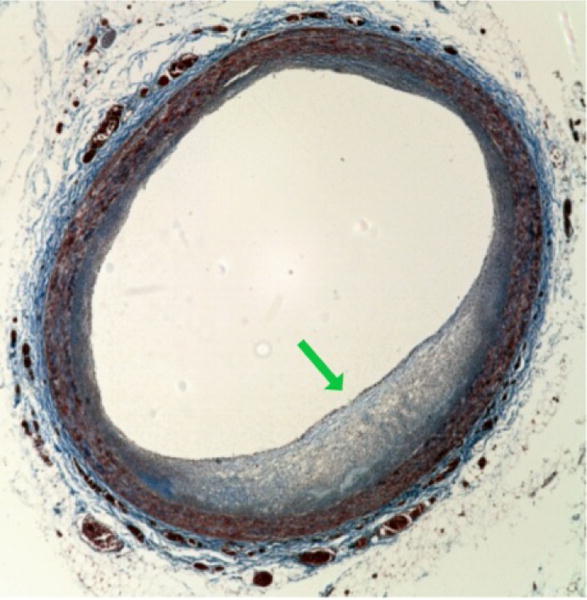
Plaque with Necrotic Core. This image shows a thin walled plaque with a necrotic core (green arrow). It can be seen it is not just a lipid collection but contains other components such as cellular debris and microvasculature (distinct from a pure lipid collection). OCT imaging work has been focused on lipid collections and not necrotic cores.

Part of the confusion lies in the fact, as will be discussed, that the ‘diffuse boundary’ criteria currently used to identify ‘lipid’ plaque is insufficient. Further leading to the confusion is that what need to be identified based on histopathological studies of ACS is necrotic cores, but OCT investigators have been looking predominantly for lipid cores (which is made worse by the literature incorrectly using the terms necrotic and lipid interchangeably). A necrotic core (as opposed to a lipid collection) contains a large amount of cellular debris, cells, and microvessels, in addition to lipids, particularly free cholesterol (example in Figure 1). From Figure 1, it can be seen that unlike pure lipid, there are a large number of scattering elements within the core. This paper attempts to clarify misconceptions that current OCT techniques can reliably identify TCFAs and provides alternative OCT approaches to achieve this objective.

Before discussing the basis of the belief that OCT can identify necrotic plaques, a brief overview of OCT is given. OCT is analogous to ultrasound, measuring the backreflection of infrared light rather than sound [20,21,23]. OCT identifies small thin-capped plaques better than any currently available imaging modalities. Cardiovascular OCT has a resolution of approximately 20 μm, catheters less than 1 mm in diameter, and an acquisition rate faster than 30 frames/second. In the early 90’s we published the first work on OCT cardiovascular imaging, which focused on vulnerable plaque assessment [21,24]. Though we have published a large number of studies since, aspects of this paper are particularly relevant to the issues of OCT plaque characterization discussed in the current paper.

The belief current OCT approach can define core composition accurately is primarily based on one study that claimed lipid plaque (the term lipid plaque again would later incorrectly be used interchangeably with necrotic core) had a diffuse intimal lipid border (1992, Yabushita et al.) that could be used as a marker for lipid cores [25]. We have previously expressed concerns in short editorials but this paper performs a more detailed analysis [23,26–29]. As lipid does not backscatter, some criteria needed to be introduced to differentiate lipid areas from areas where signal was lost from rapid surface attenuation [23,27–30]. In other words if there was no backreflection below the intima it could be either because it was lipid (which gives no backreflection) or because light did not penetrate. The current paper argues that in OCT’s current form and how it is applied, it can’t reliably identify TCFAs because it can’t accurately identify necrotic cores. The Yabushita et al. paper claimed that lipid cores (again we are actually looking for necrotic cores) could be differentiated from signal loss by having diffuse rather than sharp intimal-core borders. There are several lines of evidence that do not support this assertion. First, these criteria only had a 70% predictive power for lipid plaque [25]. The problems should have been obvious from the author’s own observations “False-positive OCT diagnoses of lipid-rich plaques often contained histological evidence of small amounts of lipid present within a predominantly fibrous plaque. These lesions, perceived as lipid-rich by OCT, were interpreted as fibrous plaque by histopathology, resulting in a relatively low sensitivity of the OCT criteria for diagnosing fibrous plaques (71% to 79%).” Second, as will also be discussed below, there is no theoretical reason why an intimal-core interface should be diffuse. This is a point we have both argued and demonstrated before and will be discussed here. We have demonstrated (and will show in this paper) both fat cells and many lipid plaques have borders that are sharp, consistent with the fact that large mismatch in refractive index lead to sharp borders. On the other hand, when many lipid crescents or calcium deposits are present in the intima this will result in phenomena known as multiple scattering, the physics of which is described below, that will make the intima-core border diffuse. This is independent of the core composition. Third, over 2/3 of the specimens for the study were from elastic and not coronary arteries, which distinct mechanisms of rupture. The only image of a coronary plaque in that paper had no histopathology for unknown reasons. It is unclear how many lipid plaques, if any, came from coronary arteries. Fourth, the OCT readers had minimal experience, an engineer and student. Fifth, they did not look at necrotic plaque but instead looked at lipid plaque. According to the paper, the authors “classified all of the plaques in the validation set as fibrous, fibrocalcific, or lipid rich”. This is not a clinically useful categorization. Sixth, several other papers have confirmed the only limited utility of the ‘diffuse border’ criteria but it still continues to be used as the standard for identifying a plaque as having a lipid core. Similar results were obtained by a second group in 2006, but 2 additional studies performed the same year had even less promising results; one of these studies reported only a 45% sensitivity and 83% specificity for identifying lipid-filled plaques [26,31,32]. From this, along with data below, it is likely that the results of the Yabushita et al. reflect a higher incidence of intimal lipid or calcium in necrotic plaques, which lead to the intima-lipid border appearing diffuse on imaging. This makes it an indirect relationship rather than a direct measure, contributing to the relatively low sensitivity [33].

It should be noted that our conclusions are also consistent with the 2012 Consensus Standards for Acquisition, Measurement, and Reporting of Intravascular Optical Coherence Tomography Studies. The problem is these important conclusions were not stated prominently in the paper [11,34]. First the report defined “A necrotic core by OCT is a signal-poor region within an atherosclerotic plaque, with poorly delineated borders, a fast IVOCT signal drop-off, and little or no OCT signal backscattering, within a lesion that is covered by a fibrous cap.” It was then noted “At present, there are no definitive published studies directly comparing OCT lipid pool–containing plaques with necrotic core by histology, and as a result, the Evidence Level was determined to be Low for OCT delineation of necrotic core.” In other words, calling a lesion a necrotic core by OCT imaging is not based on any data in the literature. OCT’s ability to identify necrotic cores is untested to date. This is consistent with the current paper and previously expressed concerns [23,27–30]. Unfortunately, these important conclusions were not emphasized in the conclusions of the paper and most still believe that diffuse borders identify necrotic cores, an unsubstantiated conclusion we have noted numerous times [11].

From our initial paper that established OCT for cardiovascular imaging (and imaging in non-transparent tissue in general), several points relevant to the current manuscript will be raised. First, it demonstrated OCT’s ability to identify intimal caps at micron scale resolutions, which few now would dispute. Second, the reason we were able to image in non-transparent tissue was that we identified using the 1300 nm wavelength range for imaging in arteries (830 nm had been used in the eye) to more than double the penetration. At this wavelength, lipid scattering and absorption is negligible. Third, it looked at plaques with high lipid content and thin caps. We did not differentiate lipid collections from necrotic regions because the importance of this would not be clear till several years later. But of significance to this paper and illustrated below, all high lipid plaques in that paper, except for one, had well-defined and not diffuse borders (the one plaque provides insight and will be discussed below). This we will see is inconsistent with the Yabushita et al. Fourth, it demonstrated that pure lipid was transparent to the infrared light while water based tissue such as intima and smooth muscle backscattered signal. But a point which seems to be missed from that paper, but relevant to the current discussion, is that fat cells (but not pure lipid) led to rapid deterioration of the signal. This seems counterintuitive (as lipid is transparent) but it is due to the mismatches in the refractive index between the inside and outside of the cells (1.35 versus 1.55) as well as their size (this is similar to the mechanism where we demonstrated why blood has poor penetration) [21,35]. These principles would support that intima-lipid borders would generally be sharp unless the intima contained significant lipid or calcium collections. This type of intima would lead to diffuse intimal-core borders by a process known as multiple scattering [23,35]. We have discussed this most recently in a 2011 Circulation paper as well as other prior work but it will be readdressed in far more detail here [27].

It is essential to identify high risk TCFA accurately for selective intervention to prevent ACS. To do so, thin capped necrotic versus non-necrotic plaque need to be differentiated. Current OCT approaches do not accurately identify necrotic cores for the reasons described. We will demonstrate this both by discussing the underlying physics involved and as well as presenting relevant OCT images. In the discussion, we will conclude by describing potentially superior OCT approaches.

## Methods and Results

**Figure 2:**
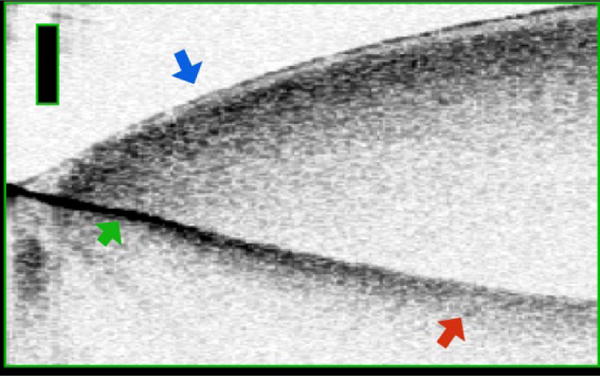
Diffuse Borders Produced by Multiple Scattering. This Figure is of a drop of blood on a metal reflector. At the edge of the blood (blue arrow) the reflector is sharp (green arrow). As the blood gets thicker the reflector appears diffuse due to multiple scattering from the large amount of blood cells (scatterers). Bar is 500 μm.

In the next few paragraphs, we will provide data that diffuse borders are caused by multiple scattering from the composition of the intima and not either lipid or necrotic cores. In this paragraph, how multiple scattering leads to diffuse borders will be discussed. OCT requires detecting only single scattering photons for accurate ranging and producing images. This is because it is measuring the time of flight. We can envision this as each single photon leaves the source, it ‘bounces directly back’ to be detected. OCT is using ‘the time of flight’ to estimate distances. However, if the photon instead of directly coming back is ‘bounced around’ or multiply scattered before coming back, the time of flight is not representative of the distance. The longer flight results in the OCT system plotting the back reflection as coming from a deeper location in the sample than its true physical position. So if we take a strong reflector, say a metal reflector, the OCT image will show blurring or signal behind the image not because it penetrates the reflector, but because of the longer time of flight from the multiple scattering (Figure 2) [36]. Though scattering theory is more complex than described here and requires the use of quantum mechanics, we can approximate what situations will lead to multiple scattering. Multiple scattering is more likely to occur when 1. Scatterers are much larger than the wavelength, 2. The refractive index mismatch between the scatterers and environment is high, 3. The shape of the scatterers deviates from spherical, and 4. The concentration of scatterers is high (this holds for red cells as in Figure 2 and it also holds for intimal lipid collections). High concentrations of cholesterol deposits or calcium clusters (but not macrophages) in the intima would therefore lead to diffuse intimal-core borders. On the other hand, there is not a theoretical basis why a core with a high concentration of lipid, but without these changes in the intima, would explain the diffuse intima-lipid interface.

**Figure 3:**
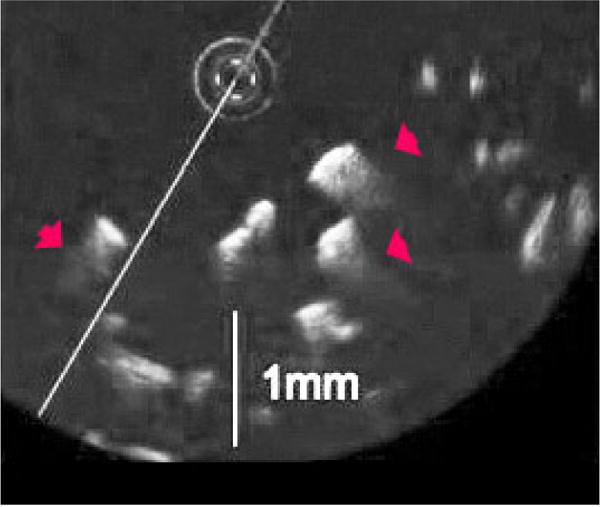
Diffuse Borders Produces from Solid Scaffolding in Air. The scaffolding is made of polylactide-co-glycolide (PLGA) and is commonly used in tissue engineering. PGLA is both a strong scatterer, is large relative to the wavelength, and has a high refractive index relative to air or saline. The red arrows in the Figure show false information (diffuse borders) distal to the imaging probe.

The data presented here either came from previous publications by our group or was data unused from those studies. For each the detailed methods can be found in the original papers. The images of scaffoldings in Figure 3 illustrate multiple scattering leading to diffuse borders when no lipid is involved [36]. The light is coming from the circular probe in the image. The scaffolding is Polylactide-Co-Glycolide (PLGA) that is commonly used in tissue engineering. PGLA is a strong scatterer, has a large relative to the wavelength, and has a high refractive index relative to air or saline, leading to multiple scattering. The red arrows in the Figure show false information (no actual structure) or diffuse borders.

In addition to the fact light doesn’t penetrate the scaffolding, it is clearly unrelated to the medium at the interface since it is the same on the proximal and distal side. High scatterers in the intima, such as lipid crescents and calcium deposits, will lead to a similar phenomenon.

**Figure 4:**
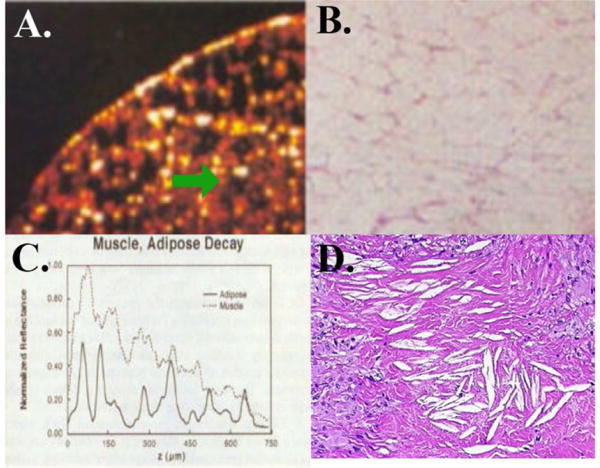
How Adipose Tissue and Lipid Crescents Lead to Multiple Scattering. In A, adipose tissue is shown where the lipid is completely transparent but there is a rapid loss of resolution and contrast with depth (green arrow). The histology is shown in B. In C, there is a rapid loss of contrast with depth relative to muscle due to multiple scattering. In D, cholesterol collections in intima are seen which have similar composition to the fat cells and result in multiple scattering.

As stated, in our initial publication describing OCT for cardiovascular imaging, we looked at the imaging of fat cells (and lipid collections) versus water-based tissue [21]. From that paper, Figure 4 shows adipose tissue (4A) and the corresponding histology (4B). It can be seen that the lipid within the cells is transparent at this wavelength. However from 4A, but also the graph in 4C, the signal in the fat rapidly deteriorates (green arrow). Differentiation of individual fat cell structure is almost gone at 350 μm. In the paper and here we included muscle for comparison in 4C that decays more slowly.

With fat, it is the large number of refractive index mismatches between the lipid and supportive tissue that leads to scattering (which is the same mechanism for blood), as well as the size of the cells, but not the lipid itself. Figure 4D shows cholesterol collections in a coronary artery intima, which have similar properties to the fat cells (high index mismatch, large irregular shape, and significant concentrations). This is why lipid collections in the intima lead to multiple scattering and diffuse borders.

**Figure 5:**
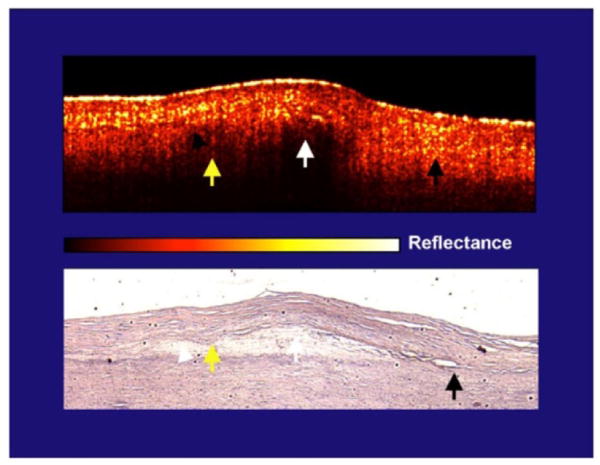
Artery with Both Diffuse and Sharp Borders Related to the Intima and Not the Core. In the same plaque, areas with a low intimal scattering had a sharp intimal-core border while areas with a highly scattering intima had a diffuse boundary whether over lipid core or the media. The yellow arrows demonstrate the cap-lipid interface that is diffuse and covered by a highly scattering intima (yellow reflections in intima). The white arrow, which is also over lipid, identifies a cap with lower scattering, and the intimal-lipid interface is sharply defined. Both are over the same lipid collection. Finally, the black arrow shows the intimal-elastic layer interface (no core present) that is diffuse with an intima that is highly scattering. This is consistent with scattering theory, but is also instructive because one core contains both diffuse and sharp borders in the same plaque.

Figure 5 is an instructive plaque [21]. In the same plaque, areas with a low intimal scattering had a sharp intimal-core border while areas with a highly scattering intima had a diffuse boundary whether over lipid core or the media. The yellow arrows demonstrate the cap-lipid interface that is diffuse and covered by a highly scattering intima (yellow reflections in intima). The white arrow, which is also over lipid, identifies a cap with lower scattering, and the intimal-lipid interface is sharply defined. Both are over the same lipid collection. The black arrow shows the intimal-elastic layer interface (no core present) that is diffuse with an intima that is highly scattering. This is consistent with scattering theory, but is also instructive because one core contains both diffuse and sharp borders in the same plaque.

**Figure 6:**
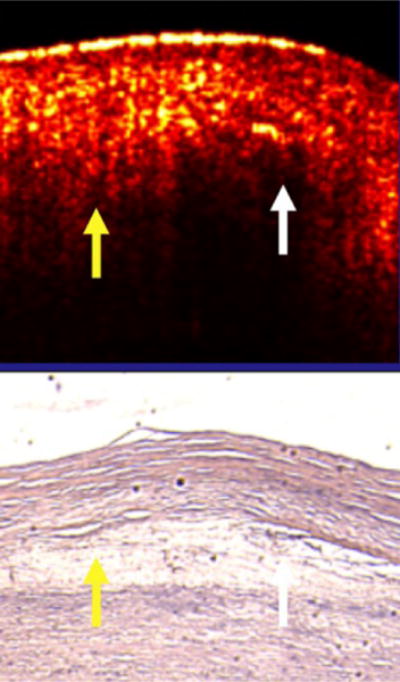
Enlargement of Figure 5. If we enlarge the area with the yellow and white arrow (Figure 6), it can be seen that the yellow arrow points to an area that contains a high concentration of lipid collections and has a diffuse border. In contrast, the white arrow has minimal collections and the border is sharp. This slide and Figure 5 contradict the conclusions of the Yabushita et al. paper.

If we enlarge the area with the yellow and white arrow, it can be seen that the yellow arrow points to an area that contains a high concentration of lipid collections and has a diffuse border (Figure 6). In contrast, the white arrow has minimal collections and the border is sharp. Figures 5 and 6 contradict the conclusions of the Yabushita et al. paper.

**Figure 7:**
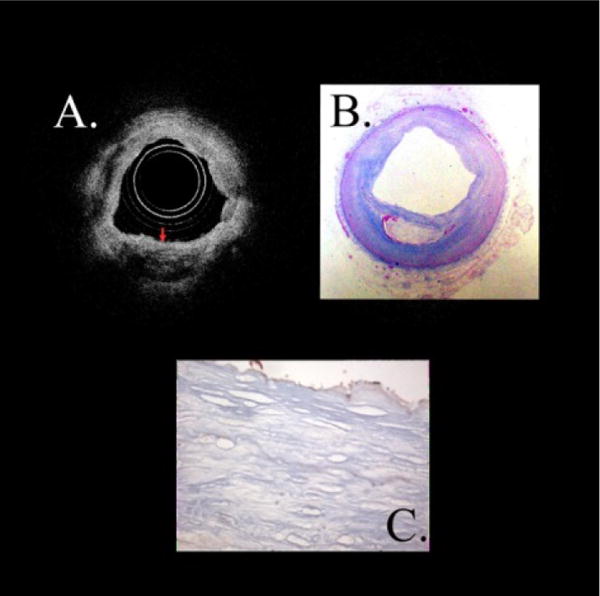
TCFA with Diffuse Borders and Many Cholesterol Collections in the Intima. In Figure 7, in the OCT image we see a necrotic core (confirmed by histopathology) where the intimal-core border is diffuse38. Increased magnification of the intima demonstrates that it has an abundance of lipid collections.

**Figure 8:**
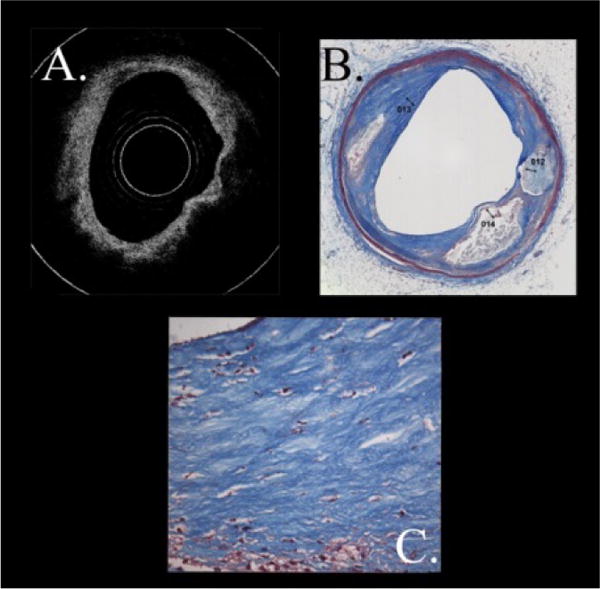
TCFA with Sharp Borders and Minimal Cholesterol Collections in the Intima. In this Figure, necrotic plaques are present as identified by the green arrows38. But the borders are sharp. Magnification of the intima demonstrates minimal lipid collections consistent with the lack of diffuse borders in spite of the plaque having a necrotic core.

Two additional examples of the correlation of diffuse boundaries with intimal cholesterol collections are shown. In Figure 7, in the OCT image we see a necrotic core (confirmed by histopathology) where the intimal-core border is diffuse [37]. Increased magnification of the intima demonstrates that it has an abundance of lipid collections. In the second example in Figure 8, necrotic plaques are present as identified by the green arrows [37]. But the borders are sharp, particularly the one to the right. Magnification of the intima demonstrates minimal lipid collections consistent with the lack of diffuse borders in spite of the plaque having a necrotic core.

## Discussion

Almost 10% of adults in the United States are in a high risk group for ACS at >2% per year [3]. As stated in an editorial by Eugene Braunwald, such individuals, in addition to intense global risk factor reduction, may be better served by further identification of vulnerable plaques in those who are at very high risk (i.e., >15% acute coronary events per year) [1].

Therefore the identification of TCFAs predisposed to ACS progression is one of the most aggressively researched areas in cardiology. It has been proposed that OCT, in its current form, can reliably identify TCFAs [25]. We argue that using the current criteria of diffuse borders at the intima-core interface does not reliably identify necrotic cores. We come to these conclusions based primarily on the analysis of the Yabushita et al. work (as well as associated papers), the principles of light scattering, data from our group, and the fact that all OCT work to date has focused on lipid collections rather than necrotic cores. We have previous published work pointing this misconception, though not in the detail of the current paper [27–30]. It is also consistent with the findings of “2012 Consensus Standards for Acquisition, Measurement, and Reporting of Intravascular Optical Coherence Tomography Studies”, though they choose not to emphasize this critical point in the summary of the report [34].

This paper describes a mechanistically based reason for the diffuse borders seen. The data supports they are due to multiple scattering in the intima (from high scatterers such as cholesterol collections or calcium deposits). These scatterers are likely more common in the intima of necrotic plaque, leading to the initial speculation the diffuse borders were due to the core composition rather than the intimal composition. This misconception, which has been a widely held belief in the OCT community, as seen is based on limited data of questionable validity. In our opinion this misconception has slowed down the field of plaque characterization with OCT for the last decade. In particular, it has reduced emphasis on pursuing potential superior OCT approaches such as those discussed in the next paragraph.

This paper is not suggesting that OCT can’t identify TCFA if applied differently or in conjunction with adjuvant techniques. We will suggest several alternative approaches here:

### 1. OCT Elastography (OCTE)

In our opinion OCTE is one of the most attractive approaches for identifying TCFAs. It is a reasonable postulate that determining a thin-walled plaque is structurally weak demonstrates both that the plaque has a high concentration of lipid (particularly free cholesterol) and that it is more prone to rupture. Our group, as well as others, has published work that OCTE can determine the mechanical properties of plaque [38–52]. This will not be reviewed here but reference to many of the different OCTE approaches are provided. But to date, no OCTE technique has been scalable for accurate video rate assessments, with among the most notably challenges being the tissue strain response time [51,52]. The tissue strain takes approximately 20-40 msec to plateau after stress application. At slow acquisition rates this is not an issue but at video rate (about 30 msec/frame) the strain is constantly changing in an unpredictable manner. This is a topic we have discussed elsewhere in detail and we have recently proposed a design that would overcome current limitations for in vivo application.

### 2. Spectroscopic techniques

A second alternative for detecting necrotic cores is using spectroscopic techniques that are designed to identify high lipid content. There exists a variety of approaches under investigation, where we are developing the use of quantum second order correlation photons [48,53,54]. Techniques in this category have not reached clinical viability but offer the potential of quantifying plaque lipid.

### 3. Parallel ultrasound beam

We have previously demonstrated a parallel ultrasound beam will reduce multiple scattering, the physics of which is described elsewhere [55,56]. However, while this should allow better delineation of cap thickness, it has yet to be examined for aiding in the characterization of the core.

### 4. Alternate plaque evaluation criteria

It has been our experience that simple visual inspection may be sufficient to differentiate necrotic plaque from simple loss of signal. Unlike lipid plaque, necrotic plaque contains cellular debris (among other materials) that scatter light, which can usually be assessed directly from the image. This hypothesis has yet to be tested in blinded studies. Clinicians are accustomed to assessing images with categorical rather than numerical data (it is actually the norm). Examples are assessing infiltrates on chest x-rays and grading valvular regurgitation. However, as we have previously pointed out, the engineering community, which has done much of the cardiovascular OCT work, tends to focus on numerical data (like exponential decay) or more strongly descriptive criteria (such as diffuse boundaries) rather than qualitative categorical data. Because for over a decade the diffuse boundary criteria have been used, it has likely reduced research in this area.

## Conclusion

The ability to selectively intervene on TCFAs predisposed to rupture, preventing ACS, would dramatically alter the practice of cardiology. While the ability of OCT to identify thin walled plaques at micron scale resolutions has represented a major advance, it is a misconception that it can reliably identify TCFAs. One major reason is that the ‘diffuse border’ criteria currently used to determine ‘lipid plaque’ is almost undoubtedly from high scattering in the intima and not because of core composition. A second reason is that studies need to be focused on identifying necrotic cores characteristic of TCFAs and not lipid collections as work to date has been. Numerous other OCT approaches are available which can potentially accurately assess TCFAs, but these have not been aggressively pursed. To at least some degree, we believe this has occurred because of the ‘diffuse borders’ misconception.
